# Prevalence of HCV, HBV and HIV infections in patients and staff of haemodialysis unit

**DOI:** 10.1186/1471-2334-12-S1-P74

**Published:** 2012-05-04

**Authors:** Saraswathy Palanisamy, Santhosh B Patil, K Baskaran, H Shankar Narayana

**Affiliations:** 1 Department of Microbiology, Melmaruvathur Adhiparasakthi Institute of Medical Sciences & Research, Melmaruvathur, Tamil Nadu, India

## Objective

HCV, HBV and HIV are most problematic infections in hemodialysis (HD) patients. The cause and source of infection is multiple in HD patients. Blood transfusion, contaminated equipment and patient to patient transmission are the potential source of infection. Our aim is to find out the prevalence of these infections in our HD centre.

## Materials and methods

This study was carried out between June to November 2011. A total of 60 end stage renal disease (ESRD) patients who are on dialysis and 15 technical staff were enrolled in cross sectional study to determine prevalence, risk factor and consequences of HCV infection. Serum samples were tested for HCV, HBV and HIV antibodies using immunochromatographic test. Subsequently anti HCV positive samples analysed with third generation anti HCV test-ECLIA.

## Results

Prevalence of anti-HCV was 8.33% (5/60) and HBsAg was 1.66% (1/60). All three serological markers were negative in staff and HIV is non reactive in HD patients. HCV infection has correlation with male gender, long term HD and units of blood transfusion. Overt liver disease rarely occurs in patients with ESRD. Chronic liver disease with elevated liver enzymes, were detected in 40% of HCV patients. Level of antibody response was poor in HCV patients range from 1.192-2.064.

## Conclusion

The prevalence of HCV and HBV infections are lower in our set up. It can be attributable to undertaking universal precautions, early vaccination, anti viral therapy and isolation of infected patients. It requires stringent adherence to all precautions to decrease the infection rate.

